# Extended Endoscopic Endonasal Transplanum and Transdorsum Sellar Approach for the Resection of Retroinfundibular Craniopharyngioma With Two-Piece Dural Opening: A Technical Case Report

**DOI:** 10.7759/cureus.51850

**Published:** 2024-01-08

**Authors:** Maruf Matmusayev, Gayrat M Kariev, Ulugbek Asadullaev, Kazuhito Takeuchi, Yuichi Nagata, Hideo Harada, Ryuta Saito

**Affiliations:** 1 Department of Neurosurgery, Nagoya University Graduate School of Medicine, Nagoya, JPN; 2 Department of Skull Base Surgery, Republican Specialized Scientific and Practical Medical Center of Neurosurgery, Tashkent, UZB

**Keywords:** posterior clinoidectomy, pituitary function, dural reconstruction, transdorsum sella approach, retroinfundibular craniopharyngioma

## Abstract

The surgical treatment of retroinfundibular craniopharyngiomas is challenging due to their location and the surrounding neurovascular structures. In this report, the transdorsum sellar approach with posterior clinoidectomy, the efficacy of direct cyst puncture, and the suitability of a two-piece dural opening are presented. A 56-year-old male with visual and cognitive disturbances was referred to our hospital. Preoperative CT and MRI demonstrated a mostly cystic lesion with calcifications in the suprasellar and retroinfundibular areas. The imaging findings were suspected craniopharyngioma, and an extended endoscopic endonasal transdorsum sellar approach with posterior clinoidectomy was performed for direct access to the lesion. Two pieces of the dura were opened to prevent postoperative CSF leakage. The patient’s postoperative course was uneventful. The endoscopic transdorsum sellar approach gives direct access to the posterior cranial fossa. A direct puncture of the cyst without CSF drainage is helpful for large cystic lesions. A two-piece dural opening is easy to suture and can reduce the chance of postoperative CSF leakage.

## Introduction

The surgical treatment of large retroinfundibular craniopharyngiomas remains challenging because of their location and the surrounding vital neurovascular structures. Various transcranial microsurgical approaches have been reported [1-6].

In recent years, extended endoscopic transsphenoidal surgery has become widely performed for craniopharyngiomas. However, tumors located in the retroinfundibular or petroclival region are difficult to access via endoscopic endonasal approaches because of the narrow surgical corridor and the surrounding vital neurovascular structures. The dorsum sellae (DS) and posterior clinoid(s) create a natural anatomical barrier, which limits access to this area [2,4,5,7,8].

The endoscopic endonasal transdorsum sellar approach can be used as an alternative to the transcranial approach for tumors in these areas, offering a more direct approach, a wider surgical corridor, and a better view without brain retraction [5,7-10]. The large bony window and large dural opening provide a good surgical corridor and make the subsequent removal procedure easy, but the risk of postoperative CSF leakage significantly increases.

Consequently, adequate skull base reconstruction is important to prevent postoperative CSF leakage. Various reconstruction techniques for the skull base have been published [11-13]. Due to the shape and anatomical complexity of the dura located on the sellar floor, dorsum sellae, and posterior clinoid, it is challenging to achieve watertight dural closure even using these techniques. To resolve this problem, we created separate incisions on the clival and planum sphenoidale dura without opening the dorsum sellae dura to access the petroclival and suprasellar areas.

In this report, we describe the case of a patient with a cystic retroinfundibular craniopharyngioma who underwent an extended endoscopic endonasal transplanum transdorsum sellar approach with a two-piece dural opening.

## Case presentation

**Figure 1:**
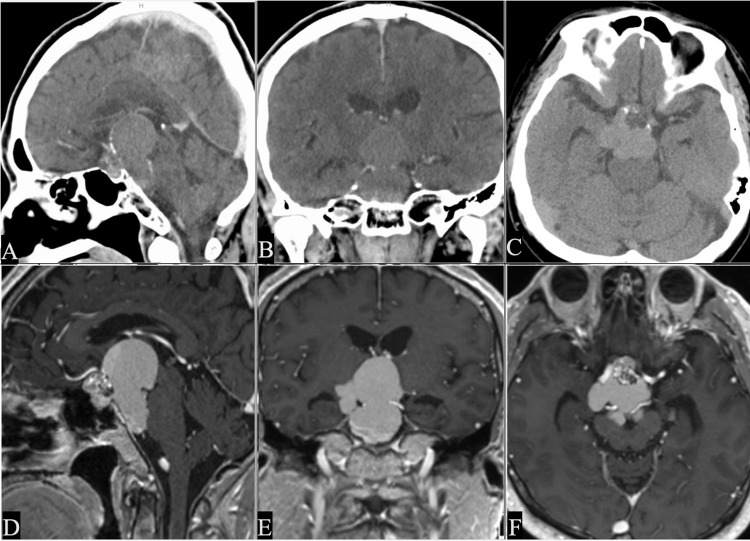
Preoperative CT and MRI (A-C) Preoperative CT and MRI imaging demonstrates the cystic lesion with calcifications suprasellar and retroinfundibular region. (D-F) Gadolinium-enhanced MRI shows a slightly heterogeneously enhanced area within the lesion, which was compressing the optic chiasm, third ventricle, and brainstem

A 56-year-old male was referred to our hospital with visual disturbances and cognitive dysfunction for the last 12 months. His cognitive dysfunction worsened during this period. A visual field test showed left homonymous hemianopia. The laboratory study found that pituitary hormone levels were within normal limits. Preoperative CT and contrast-enhanced MRI demonstrated a cystic lesion with a maximum diameter of 48 mm and calcifications in the suprasellar and retroinfundibular areas (Figure 1A-1F). Based on the imaging findings, a craniopharyngioma was suspected, and an extended endoscopic endonasal transdorsum sellar approach with posterior clinoidectomy was chosen as an option for direct access to the lesion.

Surgical procedures

Under general anesthesia, the patient was placed in the supine position with his head fixed to the Sugita 4-point head frame, slightly rotated to the right (Mizuho Medical Innovation, Tokyo, Japan), and his upper body elevated 15 degrees.

A submucosal-transseptal uninostril approach was performed under the visualization of the endoscope (EndoArm®, Olympus, Tokyo, Japan) through the right nostril. After wide sphenoidotomy, the sellar floor, tuberculum sella and planum sphenoidale, and then the upper clivus were drilled out. Removal of the sellar floor bone allowed mild pituitary gland elevation extradurally and made it easy to access the dorsum sellae and posterior clinoid processes (PCPs) [5,14].

For posterior clinoidectomy and dorsum sellae resection, we used an angled endoscope (30-degree endoscope). It provides a direct "looking-up" visualization and allows for a wider surgical space for the drilling of PCPs from the bottom to the top [8,15,16].

**Figure 2:**
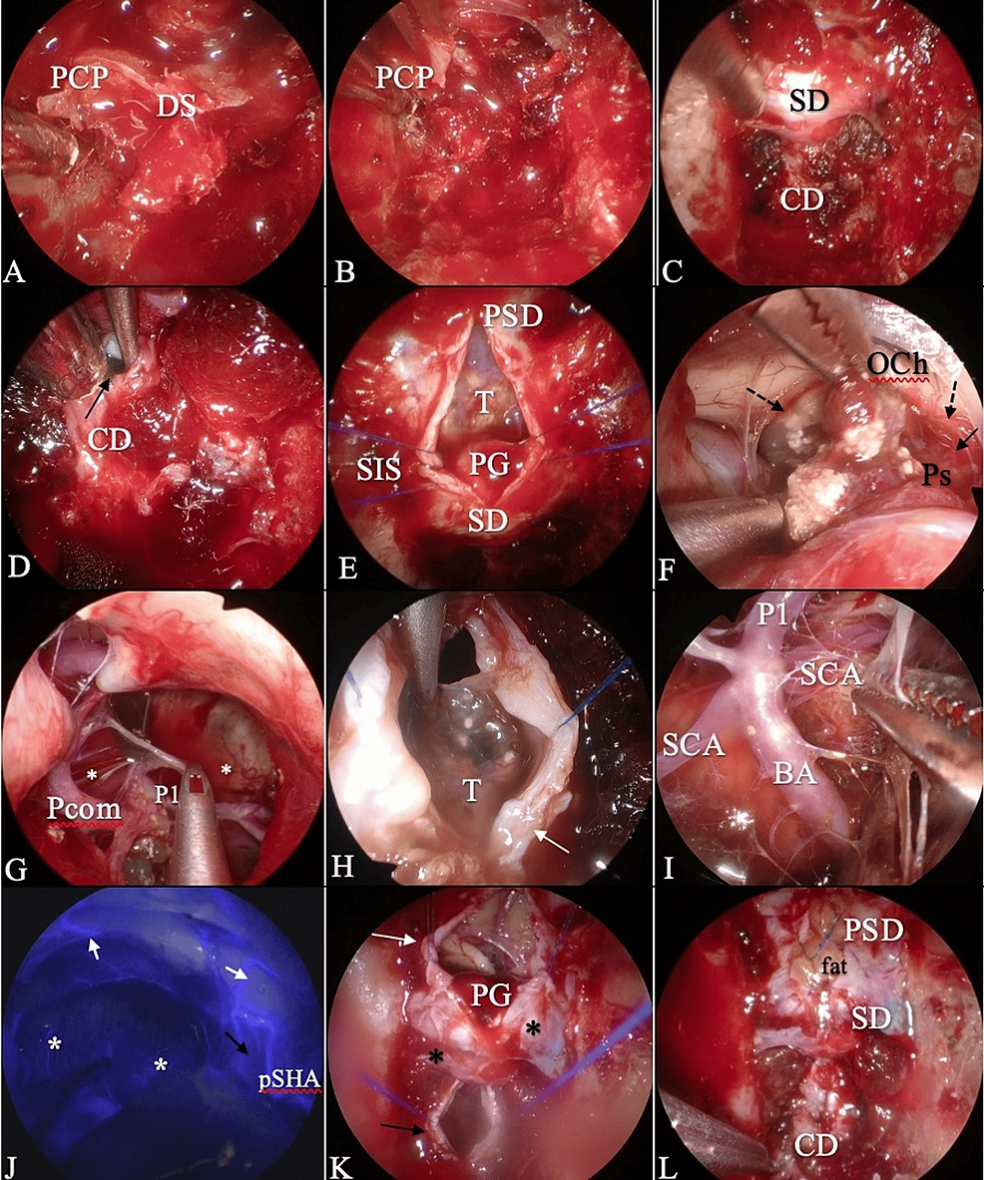
Intraoperative findings (A-C) Dorsum sellae and posterior clinoid removal. (A) Dorsum sellae and posterior clinoid detached from the attachments. (B) Removal of the posterior clinoid process. (C) Exposure of the planum, sellae, and clival dura after complete bone removal. (D) A small incision was made on the clival dura for a direct cyst puncture (black arrow). (E) Then, a linear dural incision was made from the planum sphenoidale until the sellar floor. Suturing of the SIS for hemostasis and dural tenting was performed to expand the surgical window. (F, G) Tumor removal. (F) The tumor capsule was meticulously dissected from the optic chiasm, pituitary stalk, pSHA, and (G) Pcom and perforating arteries and vital neurovascular structures. (H) The incision of the clival dura (white arrow) has been extended to remove the tumor from the prepontine cistern. (I) Detachment of the tumor capsule from neurovascular structures. (J) Indocyanine green fluorescence imaging after resection of craniopharyngioma. The white arrow indicates the pSHA Cb, the black arrow indicates the pSHA Ib, and the asterisk indicates the aTPA. (K) The intraoperative view of the two-piece dural opening after removal of the tumor. The asterisk (*) indicates the reserved dura on the sellar floor and dorsum sellae. The black arrow indicates the clival dura and the white arrow indicates the planum and tuberculum sellae dura. (L) The dural closure using the shoelace suturing technique with a fat graft and collagen matrix DS: dorsum sellae, PCP: posterior clinoid process, SD: sellar dura, CD: clival dura, SIS: superior intracavernous sinus, PSD: planum sphenoidale dura, T: tumor, PG: pituitary gland, Pcom: posterior communicating artery, PS: pituitary stalk, OCh: optic chiasm, pSHA: primary superior hypophyseal artery, dotted arrow: chiasmatic branch (Cb), arrow: infundibular branch (Ib), P1: posterior cerebral artery, BA: basilar artery, SCA: superior cerebellar artery, aTPA: anterior thalamoperforating artery

First, the dorsum sellae and upper/middle clivus were drilled until they became eggshell-thin and removed with Kerrison rongeurs. Additionally, the PCP was drilled and mobilized. After the petroclinoid and interclinoid ligaments were detached, a posterior clinoidectomy was performed using grasping forceps (Figure 2A-2E). During the drilling and removal of the bone in this region, venous bleeding from the cavernous sinus and venous plexus was controlled by hemostatic materials such as surgicel nu-knit with thrombin.

We made a two-piece separate dural opening for the clival and planum sphenoidale dura without opening the sellar floor and dorsum sellae dura. First, we made a small linear incision on the clival dura (Figure 2D) for direct puncture of the cyst and aspirated fluid content without CSF drainage, as well as performed internal tumor decompression and shrinkage of the laterally extended cystic compartment.

Then, a linear dural incision was made on the planum sphenoidale until the sellar floor. The tumor was found and dissected from the arachnoid. The pituitary stalk (PS) was located on the left side. The suprasellar portion of the tumor was removed after meticulous dissection from the optic chiasm, the PS, the superior hypophyseal arteries (SHA), and other neurovascular structures (Figure 2E-2G). Then, to remove the tumor from the prepontine cistern, an incision of the clival dura was extended until the dorsum sellae dura (Figure 2H). At this moment, the dura on the sellae was not incised. The tumor was dissected from the perforating arteries and vital neurovascular structures (Figure 2I). Gross total resection was achieved with the preservation of neurovascular structures such as the PS, SHA, posterior communicating artery, and perforating vessels. Indocyanine green fluorescence imaging (12.5 mg IV) was used to confirm the blood flow of the surrounding arteries and their perforators (Figure 2J).

After removal of the tumor, the two-piece dural opening was separately closed with a collagen matrix (DuraGen®; Integra LifeSciences, Plainsboro, NJ, USA), and an abdominal subcutaneous fat graft (Figure 2K-2L) was placed intradurally (an inlay graft) and then continuously sutured with a shoelace technique [17]. We placed another fat graft epidurally as an onlay graft and then performed rigid fixation with an absorbable fixation mesh (LactoSorb; Medical U&A, Inc., Osaka, Japan). Vascularized nasoseptal flaps were not used, and lumbar drainage was not performed (Video 1).

**Video 1:** Surgical nuances of extended endoscopic endonasal transplanum and transdorsum sellar approach for the resection of retroinfundibular craniopharyngioma with two-piece dural opening

**Figure 3:**
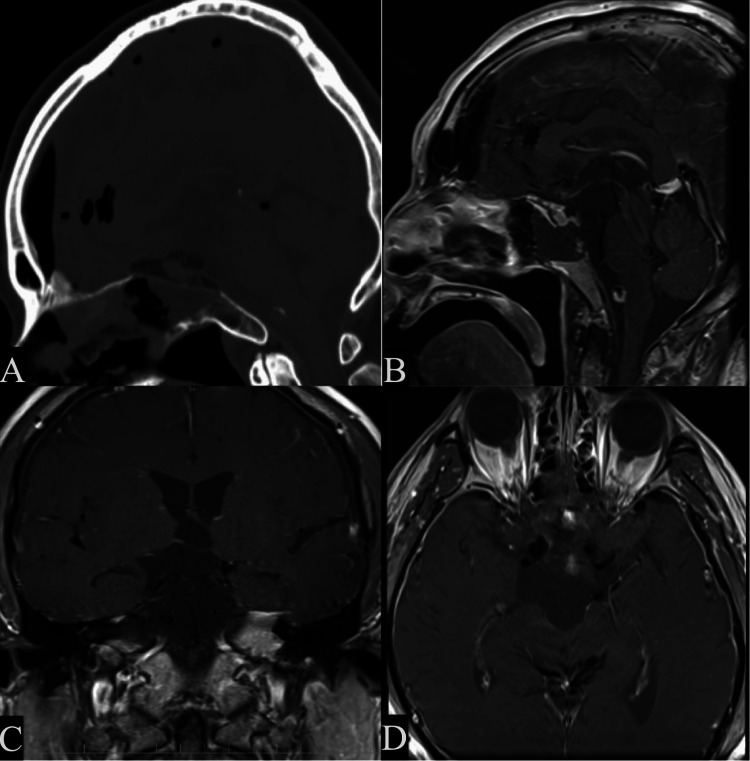
Postoperative CT and MRI images (A) A sagittal CT image demonstrates adequate bone removal from the planum sphenoidale, sellar floor, and dorsum sellae in the endoscopic endonasal transplanum and transdorsum sellar approach. (B-D) Postoperative contrast-enhanced MRI demonstrating total resection of the craniopharyngioma

The patient did not experience CSF leakage. The patient’s cognitive and visual disturbances improved immediately after surgery. Anterior pituitary functions were completely preserved. Mild diabetes insipidus was observed after the surgery and was treated with oral antidiuretic hormone vasopressin administration. The histopathological diagnosis was adamantinomatous craniopharyngioma. A postoperative CT and MRI with and without gadolinium enhancement demonstrated gross total resection of the tumor (Figure 3A-3D). During the 19-month postoperative follow-up period, there was no recurrence.

## Discussion

Posterior clinoidectomy

Kassam et al. reported endoscopic endonasal intradural pituitary transposition for a transdorsum sellae and posterior clinoid resection for tumor removal in the retroinfundibular region and interpeduncular cistern [9]. However, intradural transposition of the pituitary gland can cause decreased pituitary function due to impaired venous drainage [7,8]. Fernandez-Miranda et al. proposed endoscopic endonasal transcavernous posterior clinoidectomy with intradural pituitary transposition for skull base tumors without intradural pituitary transposition [7]. The disadvantages of this approach are massive venous bleeding from the cavernous sinus and sacrifice of the inferior hypophysial arteries for posterior clinoidectomy, which can be a cause of posterior pituitary dysfunction [7,8]. Extradural dorsum sellae resection with posterior clinoidectomy has also been described by other authors [4,5,7,8,14].

Our surgical technique for dorsum sellae resection and posterior clinoidectomy does not require pituitary gland transposition. After bone drilling, the dura mater of the tuberculum sellae and the sellar floor is completely exposed. This enables the pituitary gland to be slightly elevated extradurally, providing access to the dorsum sellae and PCPs without the necessity of pituitary gland transposition. Additionally, a 30-degree endoscope provides a direct “looking-up” view and a wider surgical space for drilling the dorsum sellae and PCPs from the bottom to the top.

Internal decompression by direct puncture

A large cystic craniopharyngioma can cause obstructive hydrocephalus due to obstruction of the foramen of Monro. In such cases, endoscopic transventricular cyst fenestration is recommended for resolving hydrocephalus [18,19]. Cyst fenestration also leads to cyst shrinkage and can reduce the surgical risks of subsequent removal surgery. However, in this case, the patient did not suffer from hydrocephalus. MRI findings revealed a large cystic craniopharyngioma with narrow ventricles. Therefore, transventricular cyst fenestration could not be adopted. However, the cystic component in the posterior fossa was attached to the clival dura. It allowed direct access to the cyst without CSF drainage.

Direct puncture of the cyst and aspiration without CSF drainage can lead to the cyst naturally shrinking with intracranial pressure. While the cyst shrinks, the tumor attachment to the surrounding vital neurovascular structures markedly decreases. It helps smooth the dissection of the tumor and its removal from vital structures. A direct cyst puncture without CSF drainage is effective for large cystic craniopharyngiomas without hydrocephalus that attach directly to the dura, which is accessible through the sphenoid sinus.

Preservation of anterior pituitary function

The inferior hypophyseal arteries are usually sacrificed for pituitary transposition. Additionally, some clinical studies have shown that bilateral sacrifice of the inferior hypophyseal arteries does not cause pituitary dysfunction in the majority of patients [20], but in some cases, anterior and posterior pituitary dysfunction was observed [21]. The sacrifice of the inferior hypophyseal arteries and subdural transposition of the pituitary gland impair venous drainage and blood supply to the gland. This can be avoided by slightly elevating the pituitary gland extradurally. By utilizing a two-piece dural opening, the dura mater of the sellar floor remains intact, and we can preserve the anatomy of the pituitary gland and the sellar region. This technique may reduce the risk of pituitary gland mobilization and anterior pituitary dysfunction.

The blood supply to the PS and the optic chiasm is provided by the SHA and its perforators, which must be preserved during craniopharyngioma surgery [16,22]. Our previous study has shown the importance of preserving the SHA infundibular branch (Ib) to maintain postoperative anterior pituitary function in craniopharyngioma surgery. If it is possible to preserve the PS during surgery, we should also take care to preserve the SHA Ib to reduce the risk of postoperative panhypopituitarism [23].

Advantages and limitations of two-piece dural opening for postoperative CSF leakage

Postoperative CSF leakage is one of the major complications of endoscopic transsphenoidal surgery, especially in the transdorsum sellar approach with posterior clinoidectomy. Adequate skull base reconstruction is important to prevent postoperative CSF leakage and associated life-threatening complications. Several reconstruction methods of the skull base have been reported to prevent this complication, including a gasket seal [13], a vascularized nasoseptal flap [11], and dural suturing [12,17,24]. Even with these techniques, it can be challenging to achieve a watertight dural closure due to the shape and anatomical complexity of the dura located on the sellar floor, dorsum sellae, and posterior clinoid. By applying the two-piece dural opening technique, the dura behind the sellar floor was not cut, the dural window was simplified, and it was made easy to close, which led to robust skull base reconstruction. As a result, we could prevent postoperative CSF leakage without the use of a nasoseptal flap or lumbar drain. A two-piece dural opening can be adapted to cystic or soft consistency lesions in the retroinfundibular area. In cases where the tumor is not cystic or not a soft consistency, we consider it necessary to open the dura more extensively to safely facilitate the removal of the tumor.

## Conclusions

The endoscopic transdorsum sellar approach with posterior clinoidectomy gives direct access to the prepontine and interpeduncular lesions. A direct puncture of the cyst without CSF drainage causes natural shrinkage of the cyst by intracranial pressure, making it easy to remove the tumor. A two-piece dural opening is easy for skull base reconstruction, which can reduce the risk of postoperative CSF leakage.
